# The Ethics and Practice of Periviability Care

**DOI:** 10.3390/children11040386

**Published:** 2024-03-23

**Authors:** Alex C. Vidaeff, Joseph W. Kaempf

**Affiliations:** 1 Division of Maternal-Fetal Medicine, Department of Obstetrics and Gynecology, Baylor College of Medicine, Houston, TX 77030, USA; 2 Texas Children’s Hospital Pavilion for Women, 6651 Main Street, Suite F1020, Houston, TX 77030, USA; 3 Women & Children’s Institute, Providence Health System Oregon, Portland, OR 97232, USA; joseph.kaempf@providence.org

## 1. Viability Is an Imprecise Concept

Since the 1960s, the gestational age at which premature infants typically survive has decreased by approximately one week per decade [1]. Survival is now reported at remarkably early gestational ages. In 2022, the Vermont Oxford Network reported neonatal intensive care unit (NICU) survival rates of 21%, 46%, and 63% at 22, 23, and 24 weeks’ gestation [2]. Presently, in industrialized nations, the so-called periviability interval refers to the gestational age period of possible survival that extends from approximately 22 to 25 weeks’ gestation. Newborns born within this period are distinguished by their need for extraordinarily complex technologic and pharmacologic interventions in order to survive. Periviability is a fluid time period of technologically assisted potential survival. Since their viability is defined as a potentiality rather than a fixed attribute, it is difficult to establish precisely either a gestational age below which the probability of survival is near zero, for which palliative comfort would be indicated, or an upper limit, above which NICU care should be universal.

Historically, gestational age boundaries have been based upon mortality and morbidity data [3,4], which are both independently influenced by birthweight and gestational age. Most published reports provide these data per gestational age weeks because, for the purpose of antenatal decisions, the birthweight is unknown. But the reported gestational age is mostly an estimate in the absence of assisted fertilization, and not more accurate than ±4–7 days at best. Furthermore, reporting these data in 7-day blocks generates further limitations: a fetonate at 23 0/7 weeks’ gestation may have different survival and morbidity rates than one born at 23 6/7 weeks, as the latter may be more similar to a 24 0/7 fetonate. Thus, both under and overestimates of the effects of gestational age make prognostication difficult. We should be cautious of using gestational age as the sole criterion when making care recommendations, because multiple factors affect survival and long-term health. Sex, birthweight, singleton versus multiple birth, exposure to antenatal corticosteroids, and maternal health conditions significantly affect the mortality, morbidity, and neurodevelopment of infants. For example, the male sex, a lower weight, multiple births, and no antenatal corticosteroid exposure may adversely impact infant outcomes [5].

Gestational age boundaries further depend on where the infant is born. Resuscitation thresholds vary between and within different countries depending upon cultural traditions, moral constructs, physician preferences, and the availability of resources. A gestational age of 23–24 weeks in a high-income country may be equivalent to 28–29 weeks in a low-income country. The remarkable variation between countries, regions, and even nearby hospitals is not limited to income categorizations. Even in similar resource environments, different culture and local practice norms generate different survival rates [6,7]. 

The care of extremely premature infants is defined by prognostic doubt and the use of high-end technology that is often experimental, unsafe, and expensive, thus necessitating a dialogic, pragmatic approach [8].Arbitrarily set limits to palliative care versus NICU admission are avoided by, within reason, embracing a collaborative recognition of the legitimacy of the “gray zone”, better phrased as the “zone of parental discretion”. There is a low end to the zone of parental discretion, where treatment would be considered by some pregnant women and families too risky and not in the infant’s nor their best interest, and a high end to the zone, where NICU care is reliably expected to be able to support a healthy child [9].Neither end should be determined by physicians alone. Instead of applying rigid rules which ignore the inherent cultural, socio-economic, and religious differences among countries, regions, and even between pregnant women within the same community, every opportunity should be sought for an informative conversation and authentic, shareddecision making [7,8,9,10,11,12].

In the US, although only 0.4% of all pregnancies are delivered at less than 26 weeks, these infants account for 40% of all neonatal deaths [9]. Extremely premature infants typically suffer major morbidity, including significant neurodevelopmental impairment, which unfortunately has not improved consistently in recent years [13,14]. Yet, the very possibility of survival at these gestational ages offers hope to families, while introducing a number of ethical questions at the same time.

## 2. Ethical Framework for Periviability

Principlism, rooted in the ethical foundations of beneficence, nonmaleficence, autonomy, and justice, serves as a major bioethical framework for the examination of moral dilemmas [15]. Beneficence (doing good) and nonmaleficence (doing no harm) are delicately balanced in extreme prematurity care. For some physicians, due to the irbelief in protecting the sanctity of life, any possibility of autonomous life should prompt all possible measures to safeguard an infant. But, based on principlism’s premises, a universally aggressive obstetric and NICU approach for all births <26 weeks’ gestation may not always be beneficial, nonmaleficent, and justified, merely because it affords the infants a small chance of survival [16,17,18,19,20].

Principlism focuses upon autonomy, perhaps at the expense of justice. Patient autonomy for pregnant women (with an emphasis upon informed, uncoerced, and individual-preference-based choice) underlies their right to make medical decisions for themselves, such as the time and mode of their delivery, fetal monitoring, and medications. The American College of Obstetricians and Gynecologists (ACOG) unequivocally advocates that physicians should never violate women’s basic right to personal autonomy and bodily integrity [21,22]. But patient autonomy is contentious under circumstances of periviability, where the patient is the fetus or the neonate and unable to exercise autonomy. Complex decisions in scenarios with uncertain outcomes and risk will require dialogue between healthcare practitioners and the pregnant woman/family, with both parties being recognized as legitimate surrogate decision-makers [3,4,20,21,22].Both parents and physicians have a moral duty to act in the best interest of the infant. But this “best interest” and its associated value judgements are ill-defined and understood differently by different individuals [23]. An extremely premature infant’s best interest is inherently ambiguous, an incoherent, even unknowable standard [8,9,10,11].When is the “best interest” of any newborn death? When is the infant’s “best interest” life, but with significant chronic health problems and impaired neurodevelopment? [19,24,25].

A critical distinction is drawn by the availability of interventions reliably expected to benefit the infant. Parents have an ethical obligation to authorize clinical interventions that are reliably expected to benefit the infant (and later child). When NICU treatments are clearly beneficial, the infant’s right to NICU care outweighs the parents’ surrogate authority to refuse care. Yet even this is contentious; e.g., vaccine refusal in children is growing more common in the US. When, on the contrary, there is uncertainty about the expected benefit of interventions, the parents’ obligation to acquiesce to physician recommendations decreases and their authority to refuse, as a surrogate, increases (the zone of parental discretion).

In the US, the professional guidelines from the ACOG and the American Academy of Pediatrics (AAP) recommend that the management of pregnant women at less than 26 weeks’ gestation always be guided by shared decision-making, compassionate dialogues, and informed consent [3,4,21,22]. The crux of this problem remains the inconsistent application of these guidelines.

There are countries (Brazil, France, Malaysia, Mongolia, Norway, Sweden, and Taiwan) where the majority of physicians believe that resuscitation decisions best reside with them, with parents being afforded little to no involvement in perinatal decisions [6,7,11,23,26,27,28]. A paternalistic attitude is not inherently unethical nor undesirable, depending upon local culture. It may be a more compassionate model, minimizing the burden of decision-making for pregnant women and families who find it confusing, and even agonizing, to decide. 

Norwegian guidelines suggest that parents be actively shielded from the burden of responsibility [29]. Such an approach does not exclude parents from decision-making, but rather facilitates their participation in it, establishing, through compassionate communication, the extent of their desired involvement based on personal, cultural, and/or religious preferences. Parents are involved in the decision-making, but the physicians are the ones who ultimately make the medical–ethical assessment and take responsibility. A similar view is shared in Switzerland, where both physicians and nurses feel the parents cannot consistently act in their extremely premature infants’ best interests [30].

## 3. Healthcare Justice, Maternal Health, NICU Costs, and Long-Term Quality of Life

At the lower end of the zone of parental discretion, where a large majority of extremely premature infants either die in the NICU or survive with chronic illnesses and neurodevelopmental impairments, principlism’s justice pillar is contentious. Justice in healthcare is fundamentally a commitment to being truthful and fair and to an equitable distribution of benefits and harms. Aside from neonatal considerations, there are additional aspects relevant to ensuring justice. The delivery of extremely premature infants is associated with significant maternal health risks. Recent data suggest that the rate of cesarean delivery in the periviable period is increasing, to levels as high as 61% at 24 weeks’ gestation [31]. Cesarean delivery at extremely early gestations is particularly hazardous for the mother [32,33,34]. Regardless of the uterine incision type, a periviable cesarean delivery results in an increased risk of uterine rupture in a subsequent pregnancy [35].Consistent with the principles of nonmaleficence and justice, a discussion of these risks should be included in the shared decision-making. The ACOG emphasizes that the health and reproductive rights of women should not be made secondary to managing pregnancy conditions or fetal status [21,22].

The NICU is among the costliest of all intensive care units. In the US, the average 6-month expenditure for infants born at 24 weeks has been reported to be USD 603,778 [36]. The rate, timing, and nature of deaths in the NICU warrant far more scrutiny because, in parallel with the increased rate of survival during the last decades, the average time to death in the NICU has also increased [37]. Delaying ultimate death comes at great financial and emotional expense. Post-NICU hospitalizations and expenditures are also predictably burdensome, and the complex care of children with chronic health issues can adversely impact parent and sibling wellbeing [38].

Frequently, in extreme prematurity care, the discussion about long-term outcomes revolves around the idea of “quality of life”. What is understood as “quality of life” is subjective; therefore, the physician-driven dialogue concerning the quality of future life must minimize both the physician’s negative prognostic bias and unrealistic promised expectations that may never occur. The discussion remains especially relevant because the most recent reports suggest that the long-term, health-related quality of life assessments of surviving premature infants are worse than previously anticipated [39,40,41,42,43,44,45]. 

## 4. The Pursuit of Ethically Based Resolutions

When disagreements between pregnant women, families, and physicians occur, how is consensus achieved? There is often no solution free of conflicts of interest or moral subjectivity. Physicians may possess conflicts of interest related to their income, research interests, career status, personal cultural and religious biases, and the NICU census; interests often not shared by pregnant women and families. 

What other resources can assist the physician–family dialogue? Taking the disagreement from the personal value level to the legal level poses challenges, and legal action should be taken only as a last resort. In the US there have been both wrongful death and wrongful life lawsuits regarding fetonates, with no consistency in the outcome of such legal precedents. The involvement of hospital bioethics committees is preferrable; these committees function as moderators to clarify disputes, hear all viewpoints, and discuss options, but they are not designed to make extreme prematurity decisions.

Professional guidelines may also be helpful as a framework. Guidelines make implicit and explicit recommendations and, although intended to influence practice, they are, at their heart, only suggestions and in many cases vague and ambiguously worded. Not infrequently, practitioners differ from the guidelines in their practice, with personal preferences continuing to play a role. For example, more than half of Argentinean neonatologists surveyed in 2014–2015 practice in contrast to the guidelines issued by the Ministry of Health [46]. It may be that difficulties in reaching a consensus cause many countries to have no periviability guidelines. In contrast, other countries, e.g., Australia, the Netherlands, United Kingdom, and the US, have multiple sets of guidelines from different societies, which often disagree [3,4,7,11]. Moreover, because of the primacy of shared decision-making, contentious clinical situations require an individualized approach that cannot be captured in the directives of summary guidelines.

## 5. Counseling versus Dialogue

“Counseling” may not be the best word to describe authentic, shared decision-making because it assumes a physician-established hierarchy of medical–technical knowledge, and, more concerning, physicians’ moral authority [8,9,12]. Compassionate dialogue and pertinent information exchange are preferable in periviable births. Physicians listening and trying to understand pregnant women and families’ concerns, values, and beliefs is a requisite for justice.

Decisions regarding resuscitation in the periviable zone of parental discretion ideally should be made before birth and not be conditional upon the infant’s appearance at birth, which does not predict favorable or unfavorable outcomes. A novel “postponed withholding” protocol is currently being studied in Norway and may offer insights that improve extreme prematurity decisions [19]. In the not so rare occasions in which premature delivery is emergent, dating unsure, or little time is available for dialogue, most guidelines recommend resuscitation unless the fetonate is obviously below 22 weeks gestation or severely compromised [3,4,7,11,21,22].

Several other caveats characterize the shared decision-making in extreme prematurity. Since counseling (for lack of a better word) is an ongoing process, it often happens in succession between different physicians, during which message fragmentation or inconsistency should be minimized [47]. Prognosis framing that excessively presents perinatal events and outcomes as solely positive or negative should also be avoided. It has been reported that parents who received a positivelyframed prognosis (chance of intact survival) were more likely to request resuscitation than parents who received a negativelyframed prognosis (chance of death and disability) [48].

Overreliance on statistics is ill-advised. Although statistics are assumed to be objective and neutral, statistics are by their nature probabilistic, conveying uncertainty. Parents do not find statistics very useful. They want to know what will happen to their child, something we rarely can tell. There is no such thing as, say, 26% survival for an individual baby. If the child lives, its survival is 100%. If the child dies, its survival is zero. It has been reported that parents’ decisions are not influenced by quoted survival or disability statistics [49].

Regardless of the decision, it is important to assure ante- and post-natal coherence. It would make no sense to intervene prenatally if no postnatal interventions were to be offered. Incoherence may annul the moral value of prior decisions. It has been reported that, when there is no agreement between the obstetricians and neonatologists’ courses of action regarding antenatal corticosteroids, the mode of delivery, and resuscitation in the delivery room, there is a 2.4-fold increase in mortality in the first 24 h of life of extremely preterm infants [50].

Who should be the final decision-maker in cases of persistent disagreement between parents and physicians in periviability situations? About 72% of pediatricians in Malaysia would say the physician, while 57% of pediatricians in Australia feel that the parents should make the final decision [51].Acknowledging how difficult it is to find global consensus in situations where such an obstinate approach is taken, we notice that, even in countries like the US, where professional guidelines and expert bioethicists believe that the best intervention in the “gray zone” of prognostic uncertainty (periviability) should be based on the parents’ informed preferences, the translation into practice of such provisions varies, and there is no established standard of care. Among surveyed neonatologists in New England, 24% would still resuscitate in the delivery room against parental wishes, while 100% would resuscitate at the parents’ request [52]. In another survey of neonatologists in New Jersey, the rate of neonatal resuscitation against parents’ wishes was markedly influenced by gestational age: 80% at 24 weeks, 15% at 23 weeks, and zero at 22 weeks. Still, at 22 weeks, 25% of neonatologists would attempt resuscitation at the parents’ request [53].The attitude of surveyed Australian neonatologists indicated a 96% willingness to comply with parents’ wishes to withhold intensive care, despite 77% of them believing that resuscitation would have been in the infant’s best interest [54].

## 6. The Ongoing Controversy

Individual physicians, institutions, and countries differ in their approach to the decision to initiate or forgo intensive care in the periviability interval. Such variations in practice influence survival and other outcomes. The reported probability of survival from different sources may not be the maximum survival possible when all obstetric and neonatal care strategies that maximize survival are used. Caution is needed when interpreting the reported survival rates from different countries. While in the US survival is most commonly reported for births within tertiary care centers, other countries report population-based survival rates that may be lower just because they include more infants who received no or delayed intensive care. The variability in active treatment practices accounts for 75% of the variation in the survival of infants without severe impairment [55]. 

Compared to obstetricians, neonatologists are more prone to be interventional in the periviability interval in Finland, Norway, or the UK [28,56]. The opposite is reported in the Netherlands [57]. Overall, neonatal nurses are the least supportive group of aggressive maximal resuscitation in the periviability interval [58]. In contrast, medical students are significantly more prone to maximal resuscitation. One may deduce that increasing personal experience and a close relationship with the patients tend to reduce medical staffs’ desire for aggressive intervention [59].

Obstetrical and neonatal care is typically more aggressive in countries like Canada, Finland, Germany, Japan, and the US. In Japan, it is norm to attempt resuscitation in infants born at 22 weeks’ gestation, with a reported survival rate of 34% in 2005 [60]. Around the same time, survival at 23 weeks’ gestation was only 4% in Switzerland [61].The main reason for that is that, in Switzerland, in accordance with their national guidelines, only comfort care is routinely provided to babies born at 23 weeks’ gestation. If you only rarely try, only rarely will you see survival; the situation creates a self-fulfilling prophecy.

Similarly, in the Netherlands, a strict systematic policy, aimed at national uniformity and the standardization of care, limits interventions below 24 weeks’ gestation and requires parental consent for active intervention at 24 0/7–24 6/7 weeks. Some neonatologists withhold intensive care upon parental request even at 26–27 weeks’ gestation, while in other high-income countries, the initiation of intensive care at these gestational ages would be the standard of care [57].

We will conclude this discussion of periviability by accepting that it is impossible to provide a global consensus and that there can be no unifying ethical, moral, or practical strategy. Nevertheless, international dialogue should be encouraged, as some key components of ethically justified, quality care seem to persistently emerge. Decisional conflict should be recognized as inherent to human nature and made explicit [62]. The early involvement of the obstetric and neonatal team is pivotal to put forward a coherent, unconfusing, nonpaternalistic, and balanced plan of care. Following appropriate dialogue with the parents, the physicians will adjust their expectations to the local standards, local outcome data, and local availability of periviable neonatal support. 

## Data Availability

No new data were created or analyzed in this study. Data sharing is not applicable to this article.
